# Intraindividual sleep variability in young adult women with posttraumatic stress disorder symptoms and hazardous alcohol use after sexual assault

**DOI:** 10.1007/s10865-026-00695-x

**Published:** 2026-07-08

**Authors:** Alexandra N. Brockdorf, David DiLillo

**Affiliations:** 1 Department of Psychology, University of Memphis, Memphis, TN, USA; 2 Department of Psychology, University of Nebraska–Lincoln, Lincoln, NE, USA

**Keywords:** Sleep, Actigraphy, Intraindividual variability, Trauma, Sexual violence, Young adults

## Abstract

Although most research has focused on average indices of sleep, night-to-night variability in sleep (intraindividual variability; IIV) is prevalent and associated with negative health outcomes. This study examined predictors of IIV in sleep among young adult women with posttraumatic stress disorder (PTSD) symptoms and hazardous alcohol use following sexual assault, a population that may be at elevated risk for sleep variability. Specifically, PTSD symptoms, alcohol use problems, age at sexual assault, years since sexual assault, racial/ethnic identity, and sexual identity were tested as predictors of greater IIV in sleep duration, sleep efficiency, and wake after sleep onset (WASO). Participants (*N* = 82) completed baseline questionnaires and three weeks of naturalistic sleep assessment via actigraphy. Three latent random variability models examining each sleep outcome were estimated with multilevel structural equation modeling in Mplus. Less time since sexual assault predicted greater IIV in sleep duration, sleep efficiency, and WASO after controlling for all other predictors. Sexual minority women reported lower IIV in sleep duration than heterosexual women. No other predictors were significant. These findings that multiple dimensions of sleep are more irregular soon after a sexual assault highlight a potential window for early intervention. Specifically, interventions that target sleep consistency may support recovery during this time, particularly for heterosexual women who also showed greater variability in sleep duration.

Sleep is a critical component of health (Ramar et al., 2021). However, over one third of adults in the United States receive insufficient sleep duration, defined as less than seven hours of sleep per 24-hour period (Liu et al., 2016), and about one in five report difficulties with sleep continuity such as trouble falling or staying asleep (Grandner, 2019). These concerns are particularly high during emerging adulthood, which may reflect both biological and social influences on sleep (e.g., developmental circadian changes, nighttime social media use; Maslowsky & Ozer, 2014; Owens et al., 2014). Insufficient sleep is linked to a variety of negative health outcomes, including internalizing symptoms, suicidality, chronic health conditions (e.g., hypertension), and mortality (Itani et al., 2017; Liu et al., 2020; Palmer et al., 2023). Difficulty sleeping also has significant economic and occupational costs, including greater absenteeism, poorer work performance, and higher health care costs (Hui & Grandner, 2015).

Although most research has focused on characterizing nightly sleep on average, greater intraindividual variability (IIV) in sleep is increasingly recognized as an important contributor to health and is also prevalent among young adults (Bei et al., 2016; Messman et al., 2022). Sleep can vary widely from night to night due to an array of individual and socioecological factors such as psychopathology, attitudes and cognitions related to sleep, access to technology, and social and occupational demands (Bei et al., 2016; Grandner, 2018). Unfortunately, just as poor sleep on average is associated with adverse health consequences, so too is greater sleep variability. Greater IIV in sleep duration is associated with increased risk of neurological, breathing, and gastrointestinal problems, pain, and internalizing concerns (Slavish et al., 2019), as well as physiological biomarkers of inflammation (Slavish et al., 2020). In addition, older adults with insomnia demonstrate greater IIV in aspects of sleep continuity, including wake after sleep onset (WASO) and sleep efficiency, compared to those without insomnia (Buysse et al., 2010). Indeed, IIV in sleep is a core target of cognitive-behavioral therapy for insomnia (CBT-I), in which individuals are encouraged to maintain a consistent sleep schedule.

## Intraindividual variability in sleep and trauma

Despite the notable prevalence among young adults and health implications of intraindividual variability in sleep, less is known about IIV in clinical populations that are at high risk for sleep problems. Approximately 70% of people will experience at least one potentially traumatic event in their lifetime, with 6% developing posttraumatic stress disorder (PTSD; Kessler et al., 2017). Sleep disturbances are a hallmark symptom of PTSD and difficulty falling and/or staying asleep is the most commonly reported symptom of PTSD among military members (McLay et al., 2010; Pruiksma et al., 2016). Further, emerging work suggests that individuals with PTSD experience heightened IIV in sleep in addition to poorer sleep on average (Slavish et al., 2022). For instance, active duty personnel and veterans with comorbid PTSD and insomnia reported higher IIV in sleep duration and efficiency compared to those with insomnia only (Straus et al., 2015). In another study, active duty personnel with PTSD demonstrated greater IIV in sleep onset latency, WASO, and sleep efficiency but not sleep duration compared to those without PTSD (Saguin et al., 2025).

These studies provide initial support for heightened IIV across several dimensions of sleep in relation to PTSD among military samples consisting predominantly of men. However, it is less clear whether these findings generalize to other populations and/or trauma types, such as young adult civilian women who have experienced interpersonal trauma. One type of trauma exposure that may be particularly relevant in the context of poor sleep is sexual assault, defined as sexual contact that is unwanted and/or occurs without consent. Experiences of sexual assault are prevalent among young adults (Mumford et al., 2020) and may contribute to fear and avoidance of sleep, particularly when the sexual assault occurred at home or when the bedroom serves as a trauma reminder (Culbertson et al., 2001; Werner et al., 2021). Supporting this, greater sexual assault severity is associated with greater insomnia symptom severity and longer sleep onset latency, as well as lower sleep quality (Cox et al., 2023). Sexual assault is also associated with heightened risk of PTSD compared with other trauma types (Kessler et al., 2014) and insomnia symptoms are greatest among adults who have experienced sexual assault and meet criteria for PTSD (Cox et al., 2023). These findings indicate that higher rates of trauma-related distress may contribute to poor sleep after sexual assault.

Another important question involves identifying the specific characteristics that contribute to heightened IIV in the context of trauma exposure. Given emerging evidence for differences in sleep variability by PTSD diagnostic status, it is possible that other person-level characteristics also help differentiate young adults who are most at risk for high sleep variability following trauma. One such factor may be hazardous alcohol use, which has been widely linked to both PTSD and sleep difficulties. Hazardous alcohol use is elevated among those with PTSD (Debell et al., 2014) and sexual assault experiences prospectively predict drinking among young adult women (Parks et al., 2014). Further, moderate and high levels of alcohol consumption impair sleep efficiency and increase WASO in the second half of the night (Chakravorty et al., 2016), suggesting hazardous alcohol use might promote sleep variability. Indeed, hazardous alcohol use among adolescents is associated with greater IIV in actigraphy-assessed sleep offset time, as well as greater IIV across weekday and weekend sleep duration (Hasler et al., 2014; Kwon et al., 2025). Additional research is needed to determine if these findings generalize to young adult women who have experienced sexual assault.

Demographic characteristics may also be important to consider. Women tend to report higher IIV in sleep efficiency compared to men and racial/ethnic minority individuals tend to report higher IIV in sleep duration and efficiency compared to non-Hispanic/Latinx White individuals, although findings are not entirely consistent (Bei et al., 2016; Messman et al., 2022). These disparities may reflect differences in social and/or occupational roles (e.g., caregiving responsibilities) and exposure to daily stressors (e.g., discrimination) that can disrupt sleep continuity and duration. Although research has not yet examined the role of sexual identity, it is possible that sexual minority individuals could also be more susceptible to greater IIV in sleep due to disproportionate exposure to discrimination-related stressors.

## Study aims

Prior work indicates that some individuals who have experienced trauma (i.e., predominantly male samples of military personnel and veterans with PTSD) experience heightened night-to-night variability in sleep duration and efficiency (Saguin et al., 2025; Straus et al., 2015). However, it is unclear whether these findings generalize to young adult women who have experienced sexual assault or to populations with co-occurring PTSD symptoms and hazardous alcohol use, who may be at elevated risk for disrupted sleep. To address these gaps, this study examined whether key person-level characteristics predict sleep (in)stability among women in emerging adulthood who have experienced sexual assault and report co-occurring PTSD symptoms and hazardous alcohol use. This study extends prior work by using actigraphy to assess night-to-night variability in sleep duration, sleep efficiency, and WASO and by testing the unique contributions of several clinically relevant factors.

We hypothesized that the following characteristics would predict greater IIV in sleep duration, sleep efficiency, and wake after sleep onset (WASO): greater PTSD symptoms, greater alcohol use problems, younger age at sexual assault, more recent sexual assault, having a minoritized racial/ethnic identity, and having a minoritized sexual identity. Although not our primary focus, we also examined whether the same predictors were associated with mean sleep duration, mean sleep efficiency, and mean WASO. We expected that the same pattern of risk factors would be associated with shorter sleep duration, lower sleep efficiency, and greater WASO on average.

## Method

### Participants

Participants (*N* = 82) were recruited from a Midwestern city in the United States using advertisements posted online and in the community. For eligibility to participate in the larger data collection (Brockdorf et al., 2025), participants had to identity as women, be between the ages of 18–30, own a cell phone to complete data collection procedures, not be pregnant or planning to become pregnant, and have experienced sexual assault since the age of 14 as determined by endorsement of one or more behaviorally specific items assessing unwanted oral sex or penetration (Messman-Moore et al., 2010). In addition, participants were required to report multiple posttraumatic stress disorder symptoms in relation to sexual assault assessed via a score of six or more on the PTSD Checklist for DSM-5 (PCL-5; Blevins et al., 2015), and be drinking at hazardous levels assessed via a score of three or more on the consumption items of the Alcohol Use Disorders Identification Test (AUDIT-C; Bush et al., 1998). Specifically, the AUDIT-C assesses frequency of alcohol consumption, typical drinking amount, and frequency of consuming six or more drinks on one occasion. All participants were included in the current analysis.

All participants were cisgender women with a mean age of 22.8 years old (*SD* = 3.2). Approximately half of the sample (45.1%) were full-time college students, followed by 35.4% who were not students, 15.9% who were enrolled in a graduate, technical, or professional school, and 2.4% who were part-time college students. Most were non-Hispanic White (73.2%), followed by Hispanic/Latina (13.4%), Black/African American (9.8%), biracial or multiracial (6.1%), American Indian/Alaska Native/Indigenous/Native American (4.9%), Asian (2.4%), and Middle Eastern/Arab (2.4%); participants could endorse all that applied to them. Approximately half of the sample was heterosexual; 30.5% were bisexual, 4.9% were queer, 3.7% were lesbian, 3.7% were pansexual, and 1.2% were unsure.

### Procedure

Study procedures were approved by university institutional review board (IRB). Participants completed informed consent and an eligibility screening online. Eligible participants then completed online questionnaires before attending a laboratory visit. During the visit, participants reviewed informed consent, received training on study procedures, and were provided an ActiGraph wGT3X-BT actimetry sensor to wear continuously on their non-dominant wrist for naturalistic sleep assessment. Participants then completed a 21-day ecological momentary assessment (EMA) protocol involving continuous wear of the actigraph and completion of self-report surveys. At the end of the study, participants returned the actigraph and received up to $166 in compensation based on compliance with study procedures.

### Measures

#### Baseline questionnaires

##### Modified sexual experiences survey (MSES).

During eligibility screening, participants completed the Modified Sexual Experiences Survey (MSES; Messman-Moore et al., 2010). Participants first responded to 14 behaviorally specific items assessing unwanted sexual experiences occurring since the age of 14 (e.g., “Have you given in to oral sex [giving a blow job, or going down, but not intercourse] when you didn’t want to because you were overwhelmed by someone’s continual arguments and pressure?”). Next, they were asked to identify which experience was most upsetting (i.e., the index sexual assault). Follow-up questions about this index assault were administered during the baseline questionnaires. The present analyses used a single item assessing age at the index sexual assault (“How old were you when this happened?”). Because the MSES assessed experiences since the age of 14 and participants were between the ages of 18 to 30, reported age at sexual assault ranges from 14 to 30. To calculate years since the sexual assault, participants’ reported age at the index assault was subtracted from their current age.

##### Posttraumatic stress disorder checklist for DSM-5 (PCL-5).

Participants completed the 20-item PCL-5 (Blevins et al., 2015) during eligibility screening. The PCL-5 assesses past-month symptoms of PTSD, including intrusions, avoidance of trauma-related stimuli, negative alterations in cognition and mood, and hyperarousal, on a 0 (*not at all*) to 4 (*extremely*) response scale. The PCL-5 was anchored to the index sexual assault, such that participants reported symptoms in relation to their most distressing sexual assault. Items were summed for a total score, with higher scores indicating greater severity of PTSD symptoms with a possible range of 0 to 80. Prior psychometric evaluation supports high convergent and discriminant validity, as well as high internal consistency and test-retest reliability (Blevins et al., 2015). Internal consistency in the current study was high (α = 0.94).

##### Alcohol use disorders identification test (AUDIT).

Participants completed the 10-item AUDIT (Babor et al., 1992; Saunders et al., 1993) during the baseline questionnaire. Items assess patterns of alcohol consumption, as well as problems associated with alcohol use (e.g., injury, alcohol-related blackouts). All 10 items were summed for a total score, with higher scores indicating greater severity of alcohol use problems with a possible range of 0 to 40. Prio psychometric evaluation supports high validity, sensitivity, and reliability of the AUDIT (de Meneses-Gaya et al., 2009; Reinert & Allen, 2002). Internal consistency in this study was good (α = 0.77).

##### Demographics.

Participants completed items on their racial identity, ethnic identity, and sexual orientation at baseline. Regarding race/ethnicity, participants who identified their ethnicity as not Hispanic/Latina/Latinx and race as White were coded as 0; all other participants were coded as 1 to indicate a minoritized racial and/or ethnic identity. Regarding sexual orientation, participants who identified as heterosexual were coded as 0; all other participants were coded as 1 to indicate a minoritized sexual identity.

#### Ecological momentary assessment

##### Actigraphy.

Participants continuously wore an ActiGraph wGT3X-BT activity monitor and completed a sleep diary each morning during the three-week EMA phase of the study. Initial sleep metrics were derived using the Cole-Kripke scoring algorithm (Cole et al., 1992; Quante et al., 2018) in ActiLife software version 6.13.4. Adjustments were made to resolve clear discrepancies between the algorithm and activity counts and/or sleep diaries in the following scenarios if adjustments were reasonably likely to improve upon the information provided by the algorithm alone: adjusting sleep–wake time if the algorithm overestimated sleep–wake and out-of-bed times compared to the sleep diary; extending sleep periods if the sleep diary and activity counts suggested the algorithm underestimated the sleep period length; collapsing proximal sleep periods to obtain a more accurate estimate of WASO; adjusting sleep onset times if the sleep diary indicated an earlier time; deleting false sleep periods (e.g., 100% sleep efficiency and zero activity counts); or adding missing sleep periods based on available sleep diary data (Brockdorf et al., 2025). The present analyses focus on sleep duration (i.e., total sleep time), sleep efficiency (i.e., percentage of time spent asleep while in bed), and wake after sleep onset (i.e., total time awake between sleep onset and the final awakening).

### Data analysis

Descriptive statistics were conducted in SPSS and Mplus version 8.11 (Muthén & Muthén, 1998). To test primary hypotheses, three latent random variability models examining each sleep outcome were estimated with multilevel structural equation modeling (MSEM). Specifically, three models were tested examining the outcomes of: (1) IIV and average sleep duration, (2) IIV and average sleep efficiency, and (3) IIV and average WASO. Intraindividual variability in each sleep outcome was modeled as a latent random variable using the log-transformation approach (Feng & Hancock, 2024). At the within-person level, two covariates—weekend (versus weekday) and day in the study as a running count—were included and group-mean-centered to account for possible systematic variations in sleep between weekends and weekdays (Scott et al., 2025) and across the study. At the between-person level, six baseline predictors (i.e., racial/ethnic minority identity versus non-Hispanic White identity, sexual minority identity versus heterosexual identity, age at index sexual assault, time since index sexual assault, PTSD symptoms, alcohol use problems) were included. The Markov chain Monte Carlo (MCMC) procedure (i.e., Bayes estimator) was used, which is a full information approach that retains all cases. The default potential scale reduction (PSR) convergence criterion of 1.0 was used; iterations were then doubled to ensure that the PSR value remained near 1.0. For each hypothesized effect, 95% credibility intervals (CI) based on posterior distributions were examined and considered to be significant (i.e., 95% probability that the population parameter is not 0) if the CI did not contain 0.

## Results

### Descriptive results

Descriptive statistics are presented in Table 1. Approximately 32% of the sample reported a minoritized racial and/or ethnic identity and 44% reported a minoritized sexual identity. On average, participants experienced their most distressing sexual assault at age 18 and completed the study approximately 4.4 years afterwards. 11% of participants experienced their index sexual assault less than one year prior to study participation, 12.2% one year prior, 20.7% two years prior, 4.9% three years prior, 11% four years prior, 12.2% five years prior, and 28% six or more years prior. Participants reported clinically significant symptoms of PTSD and hazardous alcohol use on average based on established clinical thresholds on the PCL-5 (≥ 31–33, Bovin et al., 2016) and AUDIT (≥ 8, Saunders et al., 1993). Participants completed 1,475 nights of actigraphy assessment (85.7% of possible days in the study). Average sleep duration was within the recommended range of seven to nine hours for adults (Watson et al., 2015). Sleep efficiency was 88% and WASO was 53 min on average. Intraclass correlations (ICCs) indicated that 19% of the total variability in sleep duration, 39% of the total variability in sleep efficiency, and 42% of the total variability in WASO occurred at the between-person level; the remaining variance occurred at the nightly-level.

### Primary model results

Results from the three latent random variability models are presented in Table 2 and described below.

#### Sleep duration

Regarding sleep duration, less time since the index sexual assault predicted greater IIV in sleep duration. Contrary to hypotheses, heterosexual women reported greater IIV in sleep duration compared to sexual minority women. Racial/ethnic minority identity, age at index sexual assault, PTSD symptoms, and alcohol use problems did not predict IIV in sleep duration. Racial/ethnic minority women reported lower mean sleep duration compared to non-Hispanic White women. Sexual minority identity, age at index sexual assault, years since index sexual assault, PTSD symptoms, and alcohol use problems did not predict mean levels of sleep duration. The model accounted for 30% of the total variance in IIV in sleep duration and 28% of the total variance in mean sleep duration at the between-person level.

#### Sleep efficiency

Regarding sleep efficiency, less time since the index sexual assault predicted greater IIV in sleep efficiency but not mean sleep efficiency. Racial/ethnic minority identity, sexual minority identity, age at index sexual assault, years since index sexual assault, PTSD symptoms, and alcohol use problems did not predict IIV in sleep efficiency or mean sleep efficiency. The model accounted for 21% of the total variance in IIV in sleep efficiency and 14% of the total variance in mean sleep efficiency at the between-person level.

#### Wake after sleep onset (WASO)

Regarding WASO, less time since the index sexual assault predicted greater IIV in WASO but not mean WASO. Racial/ethnic minority identity, sexual minority identity, age at index sexual assault, years since index sexual assault, PTSD symptoms, and alcohol use problems did not predict IIV in WASO or mean WASO. The model accounted for 20% of the total variance in IIV in WASO and 16% of the total variance in mean WASO at the between-person level.

## Discussion

The present study tested predictors of intraindividual variability in sleep duration, sleep efficiency, and wake after sleep onset (WASO) among young adult women with post-traumatic stress disorder (PTSD) symptoms and hazardous alcohol use. As hypothesized, more recent experiences of sexual assault predicted greater intraindividual variability (IIV) in sleep duration, sleep efficiency, and WASO. Contrary to expectations, sexual minority women reported lower IIV in sleep duration compared to heterosexual women; however, there were no differences in IIV in sleep efficiency or WASO. Also contrary to hypotheses, PTSD symptom severity, alcohol use problems, age at the index sexual assault, and racial/ethnic minority identity did not uniquely predict IIV in sleep duration, sleep efficiency, or WASO. Findings indicate that sleep irregularity may be a hallmark of the acute post-assault period among women in emerging adulthood.

Recency of sexual assault was a consistent predictor of night-to-night variability in sleep duration and continuity across all three models, suggesting sleep regularity is compromised in the more immediate aftermath of sexual assault among young adult women. These findings are consistent with the 3P model of insomnia (Spielman et al., 1987), which theorizes that precipitating factors–such as acute stressors–can initiate sleep disruption, especially among those with predisposing risk factors that increase vulnerability to sleep disruption (e.g., genetics, temperament). Although most people recover from acute insomnia, some go onto develop chronic insomnia due to perpetuating factors that maintain sleep problems over time (Perlis et al., 2020). Consistent with this framework, our findings suggest that young adult women may experience early sleep difficulties following the highly stressful experience of sexual assault, with natural recovery occurring over time. Another possibility is that distressing reminders of sexual assault that could impair sleep regularity (e.g., bedroom serving as an assault-related cue, nightmares) are more common soon after sexual assault. However, it is notable that PTSD symptom severity did not uniquely predict IIV in sleep duration, efficiency, or WASO.

In contrast to hypotheses, women’s age at the time of the index sexual assault did not predict either IIV in sleep or mean levels of sleep duration, efficiency, or WASO. This suggests that the specific age or developmental period during which the index sexual assault occurred may be less relevant in risk for higher sleep variability than the recency of the assault. It is possible these findings would not generalize to women who experienced childhood sexual abuse at younger ages because we only recruited women who had experienced sexual assault since the age of 14. However, recent experiences of trauma help account for associations between childhood sexual abuse and adult sleep disturbances (Higgs et al., 2020), which further underscores the importance of recent trauma in shaping current sleep outcomes.

Surprisingly, neither current PTSD symptoms nor alcohol use problems uniquely predicted any of our sleep outcomes in our sample of young adult women. Regarding PTSD symptoms, it is possible that PTSD symptoms do not uniquely predict IIV in sleep once recency of the assault is taken into account. Indeed, many women experience natural recovery of PTSD symptoms following sexual assault (Dworkin et al., 2023), with rates of PTSD decreasing from 75% at the first month to 41% at the twelfth month. Similarly, alcohol use problems did not predict IIV in sleep duration or continuity, which contrasts with prior work among community samples of adolescents (Hasler et al., 2014; Kwon et al., 2025). This may be due, in part, to the nature of the measure of alcohol use problems used in the present study, which emphasizes consequences of alcohol use (e.g., difficulty meeting social or occupational roles, guilt, injuries) rather than alcohol consumption itself, and may be less directly related to IIV in sleep. Because the AUDIT-C was used to establish study eligibility, alcohol consumption was not well-suited for inclusion as a continuous predictor due to range restriction. Further, our focus on young adult women who reported hazardous alcohol use restricted variability and limited our ability to compare individuals with and without heavy alcohol use. It is possible that IIV in sleep varies based on the presence or absence of hazardous alcohol use, rather than increasing linearly with increasing alcohol use or alcohol-related problems. Finally, PTSD symptoms and hazardous alcohol use were assessed using measures that focused on symptoms in the past month (PCL-5) or past year (AUDIT). These measures may not have been sensitive to daily fluctuations that could impact IIV in sleep. In addition, more granular measures of alcohol consumption or comparable measures of other substance use across the full sample were not assessed.

Finally, most hypotheses regarding demographic differences in sleep IIV were not supported. Racial/ethnic minority women did not demonstrate greater IIV in sleep duration, sleep efficiency, or WASO compared to non-Hispanic/Latina White women, although they did show lower sleep duration on average. Lower sleep duration among racial/ethnic minority women is consistent with known sleep health disparities, which are theorized to arise from a variety of factors including discrimination, neighborhood characteristics impacting sleep (e.g., light exposure, noise, perceived safety), cultural beliefs about sleep, comorbid sleep conditions (e.g., obstructive sleep apnea), and socioeconomic status (Williams et al., 2015). Further, racial/ethnic disparities in sleep duration are greatest among women (Caraballo et al., 2022). In contrast, heterosexual women demonstrated greater IIV in sleep duration compared to sexual minority women, with no other significant differences. This finding contributes to the mixed literature on sleep health among sexual minority and heterosexual individuals, with some studies finding higher rates of short sleep duration among bisexual women and men compared to their heterosexual counterparts (Caceres & Hickey, 2020; Fricke & Sironi, 2017) and others finding no differences based on sexual identity (Chen & Shiu, 2017; Jackson et al., 2016). Further, the observed differences in IIV in sleep duration between heterosexual and sexual minority women highlight the importance of examining variability in sleep duration, not just average sleep duration, for a more comprehensive picture of sleep health among diverse young adult populations. Future studies should examine factors that may account for greater IIV in sleep duration among heterosexual women who have experienced sexual assault, as well as factors that promote more regular sleep duration among sexual minority women who have experienced sexual assault.

### Limitations

The present study focused on women in emerging adulthood, a developmental period marked by elevated risk for high sleep variability (Bei et al., 2016; Messman et al., 2022). However, this focus limits the extent to which these findings can be generalized to individuals of other genders or to those in different developmental periods, such as adolescence, middle adulthood, and older adulthood. Replication among diverse samples is critical to test whether the same key predictors emerge in populations that may be less likely to experience IIV in sleep duration and/or continuity (e.g., men, older adults). We combined diverse racial/ethnic and sexual minority identities into two categories due to small cell sizes; however, this may obscure important variability across unique identities. Although we had a large sample size at Level 1 due to repeated actigraphy data collection, future studies should include larger samples at Level 2 to bolster confidence in the present findings and improve power to detect smaller effects. Finally, we examined three important aspects of sleep related to sleep duration and continuity, which are well-captured by actigraphy, but did not assess all possible aspects of sleep, including sleep quality, timing, and alertness (Buysse, 2014).

### Future directions

Given the higher variability in sleep from night to night among women who had more recently experienced sexual assault, reliance upon average indexes of sleep may miss critical aspects of sleep-related impairment. Therefore, measures that capture nightly changes, such as daily sleep diaries and actigraphy, should be prioritized in future research with this population. The present study included women who had experienced sexual assault within the past year up to 15 years prior. Future research should focus on recruiting individuals who have recently experienced sexual assault to characterize IIV in sleep during the acute post-assault period. Longitudinal studies assessing participants in the months immediately following sexual assault could elucidate the onset of and mechanisms underlying sleep irregularity, as well as trajectories of natural recovery and/or the transition to chronic insomnia. Future studies could expand on these findings by incorporating other possible predictors of IIV in sleep, such as use of other substances (e.g., cannabis, nicotine) and mental health comorbidities (e.g., depression, anxiety). Qualitative methods may also help uncover unique reasons for and mechanisms underlying IIV in sleep after sexual assault.

### Clinical implications

These findings suggest that disruptions to sleep regularity are more common in the early years after sexual assault, highlighting an important window for prevention and early intervention. Clinicians and advocates working with young adult women who have experienced sexual assault can normalize such changes and provide cognitive-behavioral strategies for maintaining sleep regularity (e.g., setting a regular wake time, limiting time spent awake in bed, avoiding attempts to “catch up” on lost sleep). Women who experience sustained difficulties with sleep regularity may benefit from gold-standard evidence-based interventions, such as cognitive behavioral therapy for insomnia (CBT-I). Monitoring sleep variability, in addition to average sleep duration and continuity, can help determine when referral to CBT-I is warranted. Given the strong linkage between sleep and health, early intervention to enhance sleep regularity may be an important part of promoting trauma recovery among young adult women who have experienced sexual assault.

In addition to these recommendations, future clinical research could evaluate the feasibility, acceptability, and efficacy of early sleep interventions to prevent and reduce sleep disruption soon after sexual assault. For instance, single session protocols for acute insomnia (Ellis et al., 2015) could be tested to quickly address irregular sleep during the acute post-assault period. These interventions could be tested and implemented in settings that provide mental health and medical services to individuals after sexual assault, such as community and university-based sexual assault advocacy centers and emergency departments. Because greater time since sexual assault predicted lower IIV, it may also be important to examine natural recovery processes, and whether early sleep-focused interventions might support or accelerate natural recovery among women who have experienced sexual assault.

## Conclusion

Intraindividual variability in sleep is prevalent during emerging adulthood and associated with an array of negative health outcomes. In this study, we found that recent experiences of sexual assault were associated with heightened IIV in sleep duration, sleep efficiency, and WASO. We also found greater IIV in sleep duration among heterosexual women compared to sexual minority women. Interventions to improve sleep regularity among young adult women who have recently experienced sexual assault—especially heterosexual women–may be helpful to promote early and lasting trauma recovery.

## Figures and Tables

**Table 1:** Descriptive statistics

	ICC	*n (%)*	Range

Racial/Ethnic Minority Identity	-	26 (31.7%)	0, 1
Sexual Minority Identity	-	36 (43.9%)	0, 1
		*M* (*SD*)	
Age of Sexual Assault	-	18.37 (2.70)	14–27
Time Since Sexual Assault (years)	-	4.40 (3.77)	0–15
PTSD Symptoms	-	31.77 (17.57)	7–75
Alcohol Use Problems	-	8.24 (4.99)	1–27
Sleep Duration (minutes)	.19	430.05 (91.99)	110–745
Sleep Efficiency	.39	88.31 (6.67)	60.33–100
WASO (minutes)	.42	52.77 (35.06)	0–257

ICC = intraclass correlation. WASO = wake after sleep onset. Time since the index sexual assault reported in years. Sleep duration and WASO reported in minutes

**Table 2:** Primary model results for sleep outcomes

	*b*	Posterior SD	95% CI	*b*	Posterior SD	95% CI	*b*	Posterior SD	95% CI
	
	IIV in Sleep Duration (*R^2^* = .30)			IIV in Sleep Efficiency (*R^2^* = .21)			IIV in WASO (*R^2^* = .20)		

Racial/Ethnic Minority Identity	0.33	0.18	−.041, .685	0.15	0.17	−.183, .498	0.05	0.19	−.351, .408
Sexual Minority Identity	−0.35*	0.17	−.664, −.021	−0.13	0.16	−.431, .181	0.06	0.18	−.286, .403
Age of Sexual Assault	−0.01	0.04	−.085, .075	−0.05	0.04	−.124, .030	−0.06	0.05	−.144, .031
Time Since Sexual Assault	−0.07*	0.03	−.123, −.007	−0.06*	0.03	−.120, −.010	−0.06*	0.03	−.126, −.005
PTSD Symptoms	0.00	0.01	−.007, .013	0.00	0.01	−.008, .011	0.00	0.01	−.009, .012
Alcohol Use Problems	0.00	0.02	−.033, .034	−0.01	0.02	−.040, .024	0.01	0.02	−.025, .046

	Mean Sleep Duration (*R^2^* = .28)			Mean Sleep Efficiency (*R^2^* = .14)			Mean WASO (*R^2^* = .16)		

Racial/Ethnic Minority Identity	−0.57*	0.18	−.931, −.219	−1.27	1.12	−3.490, .911	2.60	5.95	−8.904, 14.513
Sexual Minority Identity	0.32	0.17	−.012, .650	−0.51	1.03	−2.469, 1.569	6.33	5.48	−4.097, 17.485
Age of Sexual Assault	0.03	0.04	−.048, .114	0.06	0.25	−.424, .535	−0.18	1.33	−2.665, 2.590
Time Since Sexual Assault	0.04	0.03	−.017, .094	0.01	0.18	−.349, .360	0.34	0.95	−1.493, 2.249
PTSD Symptoms	0.00	0.01	−.012, .008	−0.02	0.03	−.083, .040	0.10	0.16	−.225, .426
Alcohol Use Problems	0.01	0.02	−.029 .039	−0.12	0.11	−.323, .090	0.92	0.57	−.226, 2.017

CI = credibility interval.

* = significant effect. Racial/ethnic minority identity is coded as 1 (minoritized racial and/or ethnic identity) or 0 (non-Hispanic/Latina White). Sexual minority identity is coded as 1 (minoritized sexual identity) or 0 (heterosexual). All other variables are continuous. Age at the time of and time since the index sexual assault were collected via the Modified Sexual Experiences Survey (MSES). PTSD symptoms were collected via the PTSD Checklist for DSM-5 (PCL-5). Alcohol use problems were collected via the Alcohol Use Disorders Identifications Test (AUDIT)

## Data Availability

The data that support the findings of this study are available from the corresponding author upon reasonable request.
